# Pt-free, low-cost and efficient counter electrode with carbon wrapped VO_2_(M) nanofiber for dye-sensitized solar cells

**DOI:** 10.1038/s41598-019-41693-1

**Published:** 2019-03-26

**Authors:** Subashini Gnanasekar, Pratap Kollu, Soon Kwan Jeong, Andrews Nirmala Grace

**Affiliations:** 1 Centre for Nanotechnology Research, Vellore Institute of Technology, Vellore, 632014 Tamil Nadu India; 2 CASEST, School of Physics, University of Hyderabad, Gachibowli, Hyderabad 500046 India; 3 Newton Alumnus Researcher- the Royal Society London, Cavendish Laboratory, Department of Physics, University of Cambridge, Cambridge, CB3 0HE UK; 4 Climate Change Technology Research Division, Korea Institute of Energy Research, Yuseong-gu, Daejeon, 305-343 South Korea

## Abstract

The present study reports the use of one-dimensional carbon wrapped VO_2_(M) nanofiber (VO_2_(M)/C) as a cost-effective counter electrode for dye-sensitized solar cells (DSSCs); where M denotes monoclinic crystal system. Uniform short length nanofiber was synthesised by a sol-gel based simple and versatile electrospinning and post carbonization technique. The investigation of nanostructure and morphological analysis were performed by X-ray diffraction (XRD), field emission scanning electron microscope (FE-SEM), and transmission electron microscope (TEM) with EDAX. The electrochemical response was comprehensively characterized by cyclic voltammetry, electrochemical impedance spectroscopy and Tafel polarization. The electrochemical analysis of the VO_2_(M)/C nanofiber counter electrode exhibits significant electrocatalytic activity towards the reduction of triiodide and low charge transfer resistance at the electrode-electrolyte interface. The DSSCs fabricated with carbon-wrapped VO_2_(M) nanofiber CE showed high power conversion efficiency of 6.53% under standard test condition of simulated 1SUN illumination at AM1.5 G, which was comparable to the 7.39% observed for conventional thermally decomposed Pt CE based DSSC under same test conditions. This result encourages the next step of modification and use of low-cost VO_2_(M) as an alternate counter electrode for DSSCs to achieve a substantial efficiency for future energy demand.

## Introduction

More threatening next-generation energy demand and global pollution have a promising solution from renewable energy production and utilization. Among the different renewable energy sources, solar energy is regarded as a benchmark due to its abundance and advantages in more generous exploration and utilization. Dye-sensitized solar cells (DSSCs) stands out from commercial silicon-based photovoltaic devices due to its characteristic features such as flexibility, low fabrication cost, environmentally friendly, semi-transparency, easy assembling etc.^1^. More than two decades, Grätzel’s dye-sensitized solar cell has been investigated both scientifically and technically owing to its essential features and hence accepted as a viable alternative to conventional photovoltaic devices^2^. Though DSSC promises next-generation energy demand, commercialization needs more effort. Effective and significant research is undergoing in each component of the DSSC to improve its lifetime, reduce its cost and increase its efficiency^3^.

Conventional DSSC includes several components such as a dye sensitizer, a photoanode, redox electrolyte, and a counter electrode. The important component in DSSC is the dye sensitizer, which plays a significant role as a sensitizer for light absorption promoting photoexcitation to produce electron-hole pair and the foremost process in DSSC. The photoanode consists of wide band gap nanocrystalline semiconductor oxide (TiO_2_, ZnO, SnO_2_ and chalcogenides) coated conductive glass substrate (Fluorine doped tin oxide coated glass), which transmits the generated photoelectron to the external circuit. In addition to photoanode and dye sensitizer, the electrolyte also plays a vital role in driving the device and give passage for the photoexcited holes, which helps to restore the original state of the dye. The most commonly used electrolyte is the redox couple, which contains iodide/triiodide solution^4–7^. The counter electrode is the performance evaluating component in DSSC, which collects and transfers electrons from external circuit and regenerate the dye by catalysing the reduction from I_3_^−^ to I_2_. Pt-coated FTO is a promising and widely used counter electrode due to its high electrical conductivity and electrocatalytic activity towards the reduction of redox species and corrosion resistant to iodide/triiodide electrolyte etc^8^. So far, many remarkable achievements have been done in DSSC by optimizing the TiO_2_ photoanode, redox couple and Ruthenium based commercial dyes (N719, N3 dyes). However, particular interest has been drawn towards replacing the platinum counter electrode due to its high cost and availability, which hurdles the large scale commercialization of the DSSC^9^. Therefore, finding and developing an alternative to substitute Pt with all its salient features is a significant challenge and huge demand. So far, carbonaceous materials, conductive polymers, metal compounds (metal oxide, metal nitrides, metal carbo nitrides), multiple compounds and composites have been developed and tested as promising counter electrodes^10,11^. Among the above mentioned counter electrodes, some material exhibits superior characteristics like high electrical conductivity, high electrocatalytic activity, low cost and naturally abundant as compared to platinum and some materials behave inferior where still research is undertaken to improve the performance by making composites.

Since last decade, transition metal compounds including carbide, nitrides and oxides are identified as a potential candidate to substitute Pt due to their characteristic features such as low cost, thermal stability, durability, high thermal and electrical conductivity and more importantly its catalytic activity similar to platinum as reported^12^. Compared to carbides and nitrides, fewer studies are reported for oxides based counter electrodes. Among the oxide materials, WO_2_, WO_3_, SnO_2_, Nb_2_O_5_, TiO_2_, ZrO_2_, Cr_2_O_3_, MoO_2_ are attempted to study as a counter electrode for DSSC^13^. Vanadium based oxide received particular interest among researchers due to its remarkable properties attained through different stable polymorphs such as V_2_O_5_, VO_2_(M), VO_2_(B), VO_2_(A), VO_2_(D), VO_2_(BCC) and VO_2_(N)^14^. Among the different polymorphs, monoclinic vanadium oxide VO_2_(M) is receiving more attention for energy conversion and energy storage due to its unique characteristic features such as low cost compared to platinum, high energy capacity, moderate work function, and first-order metal-to-insulator phase transition at temperatures about 68 °C^15^. More reports have demonstrated that, the temperature induced phase transition facilitates appreciable change in the optical and electrical property^16^. This Transition metal oxide (VO_2_) has been identified as an unique material with different potential applications including energy storage, optical switching devices, capacitors, electro-chromic and thermo-chromic device due to its characteristic electrochemical and optical properties^17,18^. Recently, vanadium based oxides are attempted to study as a counter electrode for DSSC. In particular, vanadium-based oxide such as V_2_O_3_, V_2_O_5_ and VO_2_ (M1) nanoparticles were studied and a photoconversion efficiency of 5.40%, 1.6% and 1.25% was reported^19–21^. Until now, Geetha R. Mutta’s group has studied the performance of sphere-like VO_2_(M) nanoparticle synthesized by hydrothermal route as a low-cost counter electrode for DSSC and the reported photoconversion efficiency was 1.25%; however, the material efficiency is reported low^21^. The abundance, cost-effective and environment friendliness of the material created a room for the researchers to improve the catalytic activity of VO_2_(M) by either tuning the morphology, making composites and doping with other active materials. So far, as a counter electrode for DSSC, different synthesis routes with various morphologies have been reported. One dimensional nanostructures such as nanorods, nanobelts, nanowires and nanofibers are found to be an effective counter electrode for DSSC, as it poses large surface area and efficient charge transport due to the interconnected structure. Among the different synthesis techniques, electrospinning is a simple and versatile method to obtain well-defined 1D micro and nanostructures. Indeed, the electrospun nanofiber exhibits high surface area and high aspect ratio, which has wide potential applications. Appreciable results are reported for DSSCs with electrospun one dimensional nanostructures such as WO_2_ nanorods (7.25%)^22^, α-MoO_2_ nanorods (4.1%)^23^, FeS nanorods (6.47%)^24^. Recently, carbon coated metal oxides has been widely reported and demonstrated that the carbon in parent material improved the electrocatalytic activity, which find its potential application in lithium-ion batteries and supercapacitor^25–28^. Yifu Zhang’s group have synthesized VO_2_(M)@C core-shell structure using the hydrothermal method and improved the electrochemical performance of VO_2_(M) for supercapacitor applications^29^.

Herein, we synthesized the carbon wrapped vanadium oxide nanofiber (VO_2_(M)/C) by post calcined electrospinned nanofiber. The material has been characterized in detail and tested as a counter electrode in DSSC for the first time, which showed good electrocatalytic performance and photo conversion efficiency of 6.53%, which is comparable to the value 7.39% obtained for the conventional thermally decomposed Pt CE under standard simulated 1SUN irradiation at AM1.5 G. To the best of our knowledge, this is the highest photon to power conversion efficiency achieved for VO_2_(M) CE based DSSC employing iodide/triiodide redox electrolyte under standard test conditions. The 1D morphology and carbon wrapping over the surface has improved the photoconversion efficiency of VO_2_(M) remarkably.

## Methods

### Synthesis of VO_2_(M)/C nanofiber

The VO_2_(M)/C nanofibers were synthesized by a two-step process. In the first step, the precursor sol was prepared using vanadyl acetylacetonate as vanadium precursor, N, N dimethylformamide (DMF) as dispersing solvent and Poly(vinylpyrrolidone) (PVP) as a source of carbon and structure directing template for the growth of nanofibers. All the chemicals were purchased from Sigma Aldrich and used without further purification. The above mentioned composite sol was stirred for complete dissolution. The dissolved composite sol was heated to 80 °C for 2 hours and then cooled to room temperature. In the second step, the obtained precursor solution was transferred to 2 ml plastic syringe connected with stainless steel needle, which is to be placed in the electrospinning setup at a flow rate of 0.2 ml/hr. A positive potential of around 10–12 kV was applied between the electrode needle tip and an aluminum foil which is rolled over the rotating drum. The needle and the aluminum foil are separated at a distance of 15 cm. The generated electric field ejects out the composite jet from the needle and accelerates towards the collector aluminium foil drum, and the fibers were formed after solvent evaporation. After drying at room temperature for 2 hours, the fibers were carbonized in an air atmosphere at 400 °C for 30 min at a heating rate of 5 °C/min and then cooled to room temperature.

### Characterization

As synthesized VO_2_(M)/C was characterized by X-ray Diffractometer (Bruker D8) with Cu Kα radiation source (λ = 1.54 Å) for its crystallographic details. The morphology and structure of the as synthesized nanofiber were characterized by Field Emission Scanning Electron Microscopy (FESEM) with elemental analysis using Energy Dispersive X-Ray Spectroscopy (EDAX). High Resolution Transmission electron microscopy (HRTEM- FEI Tecnai G2S Twin) was used to further analyse the size, shape and carbon wrapping thickness over the nanofiber. Fourier Transform Infrared Spectroscopy (FTIR) analysis was performed using Perkin Elmer’s Spectrum two FTIR Spectroscopy instrument. The Raman spectrum was obtained from Horiba Scientific XploRA PLUS Raman microscope, operated with 512 nm laser. Thermogravimetric Analysis (TGA) was carried out using Perkin Elmer’s STA 8000 in the presence of air at a flow rate of 20 ml/min. X-ray photo electron spectroscopy measurements were done using ULVAC-PHI high resolution XPS with a monochromatic Al Kα (1486.6 eV) X-ray beam spectrometer with 500 µm spot size.

### Preparation of VO_2_(M)/C and Pt counter electrode

Prior to the coating, the FTO coated glass substrates (resistivity: 14Ω/sq^−1^, Sigma Aldrich) were cleaned under ultrasonication with soap solution, DI water, isopropyl alcohol and finally, dried under nitrogen blow. The VO_2_(M)/C counter electrode was prepared using a uniform mixture with 200 µl of DI water and few drops of Triton X. The paste was coated over the conducting side of the FTO glass substrate using doctor blade technique. The coated FTO were annealed at 200 °C for 30 min to remove the binder and then used as a counter electrode. Conventional Pt counter electrode was prepared with 10 mM H_2_PtCl_6_ in isopropanol solution on FTO substrate using the same doctor blade technique followed by thermal decomposition at 450 °C under air atmosphere.

### Preparation of photoanode

The cleaned FTO glass substrate was treated with 40 mM of TiCl_4_ at 80 °C for 1 hour to form a compact TiO_2_ layer. The substrate was rinsed with DI water and dried under N_2_ flow. TiO_2_ paste was prepared by stepwise mixing and grinding of 500 mg of commercial P25 TiO_2_ powder with 500 µl of DI water, 50 µl of 0.1 M HNO_3_ and few drops of Triton X using mortar and pestle. The uniform paste was coated over the conducting side of the FTO substrate and then sintered at a different temperature. First, the coated FTO was heated at 350 °C for 10 min, then 400 °C for 10 min and finally at 450 °C for 30 min. Then it was cooled to 80 °C and immersed in 0.5 mM N719 dye solution prepared with ethanol at room temperature for 24 hours. The dye-loaded photoanode was rinsed with ethanol and dried under nitrogen gas flow.

### Fabrication of DSSC using VO_2_(M)/C and Pt counter electrode

The DSSC was fabricated by sandwiching photoanode and counter electrode with a spacer in between and filled with redox electrolyte. The redox electrolyte for solar cell testing was prepared in the composition of 0.05 M I_2_, 0.5 M LiI and 0.1 M 4-tert-butylpyridine in absolute acetonitrile solution. The active area of the coated substrate was fixed as 0.16 cm^2^ for testing.

## Results and Discussion

**Figure 1:**
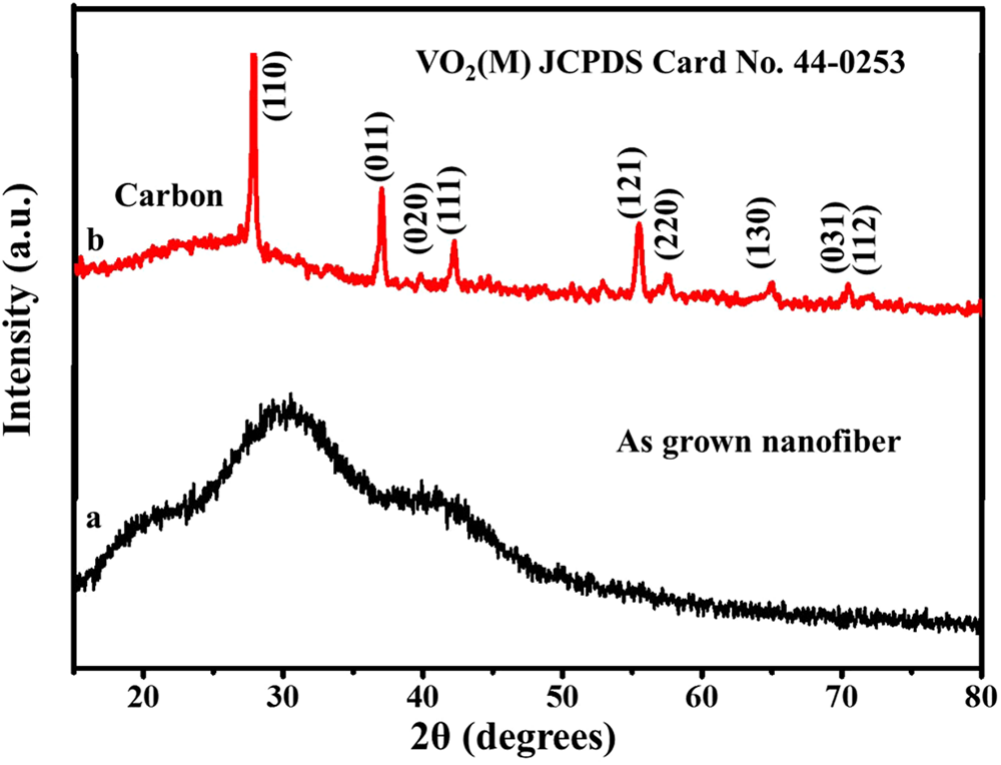
X-ray diffraction pattern of (**a**) electrospinned nanofiber (**b**) Carbonized nanofiber at 400 °C in air.

The phase composition, purity and crystallinity of the as-prepared fiber and carbonized nanofiber were examined by a powder X-ray diffraction analysis as shown in Fig. 1. All diffraction peaks of the annealed sample at 400 °C corresponds to the monoclinic crystal system of space group P21/c and can be indexed corresponding to VO_2_ (M) (JCPDS card (044-0253) with crystal parameter a = 0.57529 nm, b = 0.45263 nm, c = 0.53825 nm, and α = γ = 90°, β = 122°). No other extra peaks of other phases or impurity of other vanadium oxides like VO_2_(B), VO_2_(R) etc., was observed, indicating the high purity and good crystallinity of monoclinic VO_2_(M) nanofibers from annealed electrospinned fibers. The characteristic peak of carbon was confirmed from a broader peak from 20° to 25°. From XRD it is revealed that the as-prepared carbonized nanofiber is carbon coated VO_2_(M) nanofiber. After carbonization, all the peaks are well-defined, which suggest that the material is well formed after the heat treatment process. X-ray diffraction analysis for the VO_2_(M)/C coated FTO glass substrate that was used for photocurrent-voltage (J-V) characteristics were done and plotted in Supplementary Fig. S1. The diffraction peaks obtained shows the corresponding characteristics peaks of FTO and also the major peaks of VO_2_(M)/C. This result confirms the stability of the VO_2_(M) after testing.

**Figure 2:**
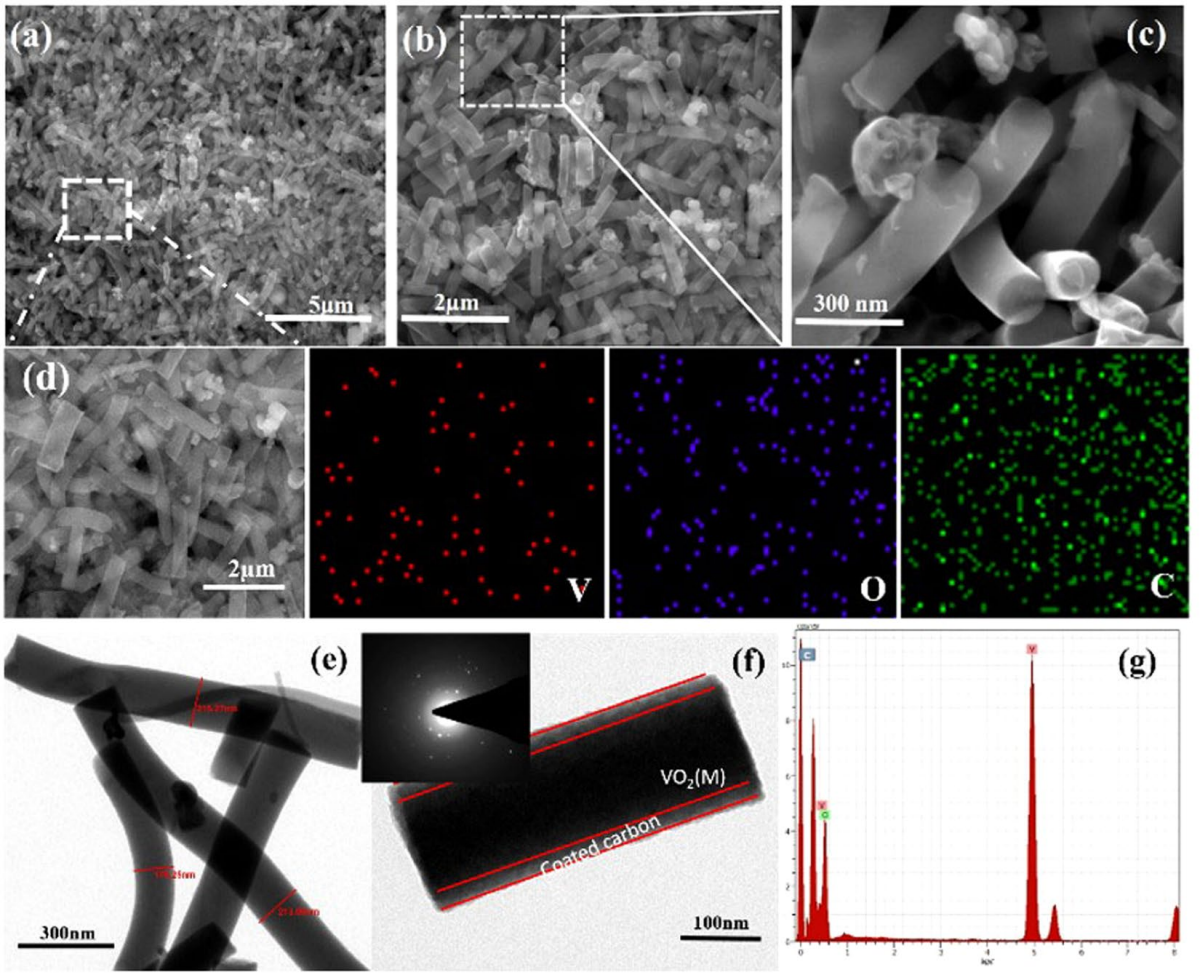
VO_2_(M)/C nanofiber, (**a**–**c**) SEM images at various magnifications (**a**) at 5 µm (**b**) at 2 µm and (**c**) at 300 nm and (**d**) Dark-field SEM image with mapping of the elements V, O and C. (**e**,**f**) TEM images at different magnifications (inset: SAED pattern for VO_2_(M)/C nanofiber) g) EDAX obtained for composite nanofiber.

**Figure 3:**
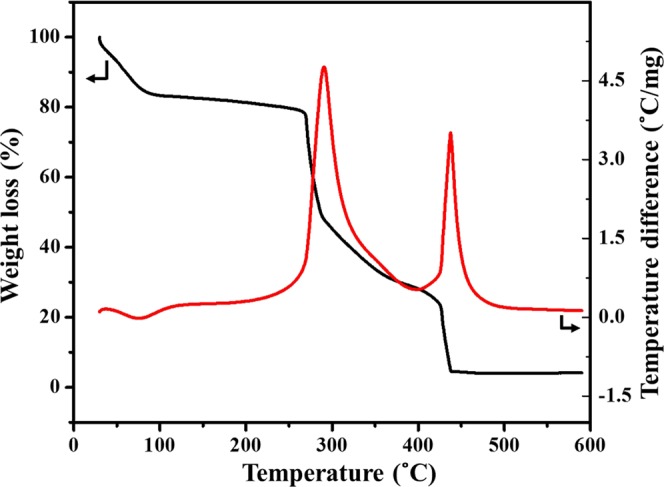
Thermogravimetric analysis of VO_2_(M)/C nanofiber shows weight loss and derivative weight loss against temperature.

The morphology of the VO_2_(M)/C nanofibers was determined by scanning electron microscope (SEM) and Transmission electron microscope (TEM). Figure 2(a–c) shows the SEM images at different magnifications of annealed electrospun fibers. The images revealed the fibrous nature of the materials synthesized and its uniformity, which results from the complete dissolution of PVP and VO(acac)_2_ to wound nanofiber. The decomposition of PVP to carbon and deformation of VO(acac)_2_ to VO_2_(M) are associated into a VO_2_(M)/C nanofiber. The as-obtained individual nanofiber was uniform in cross-section with an average diameter of around 150–300 nm and length of 1–2 µm. The bending instability of the spinning jet associates the formation of randomly oriented nanofibers. Figure 2(d) reveals compositional distribution through elemental mapping performed with SEM analysis. The dark-field image of the V, O, and C mapping of the fiber were observed to clearly understand the elemental distribution. The carbon mapping shows more intense distribution, which evidenced the carbon wrapped over the VO_2_(M) nanofiber. Detailed structural information was examined from transmission electron microscope as shown in Fig. 2(e,f), where the wrapping of the carbon layer on VO_2_(M) from the carbonization of PVP is clearly observed. EDAX analysis identifies the percentage of element composition present in fiber as given in Fig. 2g. To further analyse the formation temperature and thermal stability of the composite material, thermogravimetric analysis was done. TG-DTG analysis of the as-spinned VO(acac)_2_/PVA composite precursor fiber was done in the air to understand the decomposition mechanism of the precursor fiber and thereby define the formation temperature for the VO_2_(M)/C nanofiber. Figure 3 shows the thermogram graph with weight loss as observed in three different stages. In the first stage, 16.24% weight loss was occurred between 50 °C to 150 °C with an endothermic peak around 76.35 °C corresponding to the evaporation of moisture and solvent trapped in the precursor fibers. In the second stage, 53.14% weight loss was observed between 150 °C to 350 °C accompanied with an exothermic peak at 290.2° due to the degradation of the side chain of PVP and other organic intermediates formed during combustion. In the final stage 25.72% weight loss between 350 °C to 450 °C with an exothermic peak around 400 °C is due to the degradation of PVP main chain, oxidation of the precursor and crystallization of vanadium oxide. This indicates the formation temperature of the VO_2_(M) nanofiber. Above 450 °C, the weight of the sample remains constant indicating the complete combustion of the composite precursor fiber.

Investigation of chemical bonding between the vanadium, oxygen and carbon were analysed using FT-IR spectroscopy. Supplementary Fig. S2 illustrates the FT-IR spectra of carbon wrapped nanofibers. The vibrational bands absorbed between 1000–400 cm^−1^ represents the interaction between vanadium and oxygen. The peak around 974 cm^−1^, 730 cm^−1^ and 620 cm^−1^ corresponds to the stretching coupled vibration of V=O in oxo complexes of tetravalent vanadium. The initial vibrational band around 510 cm^−1^ and 420 cm^−1^ are a representation of V-O-V octahedral bending modes. The peaks corresponding to the interaction of vanadium and oxygen are in good agreement with the other reports^30,31^. The band around 3450–3000 cm^−1^ corresponds to the vibration of the O-H group and the stretching vibration of the oxodiperoxovanadium. The peak around 1600 cm^−1^ and 1400 cm^−1^ represents the ligand vibration from CH-N, C=O and C=C.

**Figure 4:**
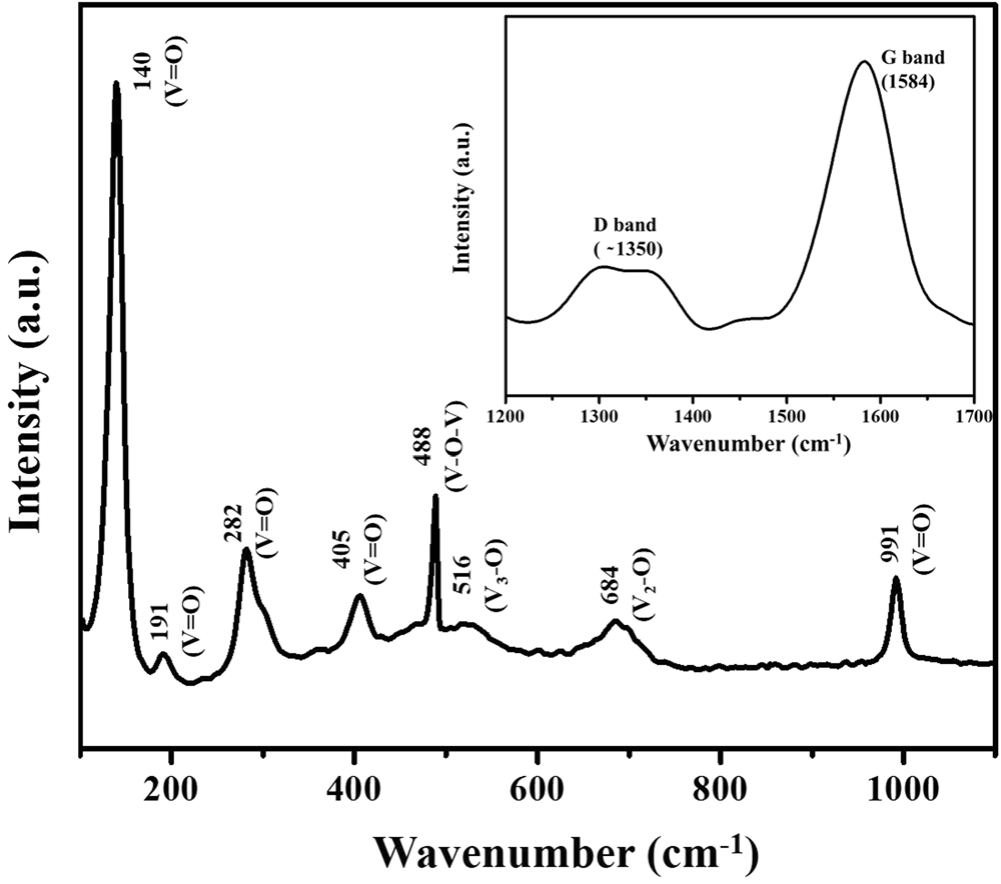
Raman spectra of the as-prepared VO_2_(M)/C nanofiber (Inset shows the Raman spectra of carbon, present in the as prepared nanofiber).

Raman spectra is a useful tool to identify molecules present over the sample. The Raman spectra of the VO_2_(M)/C nanofiber is given in Fig. 4. The prominent phonon peaks obtained between 100 cm^−1^ to 1100 cm^−1^ are the possible characteristic vibrational modes corresponding to stretching, bending and translational modes of vanadium and oxygen^32,33^. The peak at 140 cm^−1^ (B_3g_, A_g_ symmetry) and 191 cm^−1^ (B_1g_ symmetry) correspond to the bending vibration modes of the (V_2_O_2_)_n_ chain translation, which is due to the deformation of the bond between different molecular units, where the detection is strongly associated with the oxide layer structure. Moreover, the sharp and strong peak at 140 cm^−1^ indicates the long-range order in the plane of the vanadium oxide layer. The peaks at 288 cm^−1^ (B_2g_, B_3g_ symmetry) and 405 cm^−1^ (A_g_ symmetry) are associated with the bending vibration of the V=O bonds. The peak located at 488 cm^−1^ (A_g_ symmetry) is assigned to the bending vibration of doubly coordinated oxygen bridging with vanadium V-O_2_-V bond. The peak at 516 cm^−1^ (A_g_ symmetry) indicates the presence of triply coordinated oxygen V_3_-O stretching bond, which is due to the edge shared oxygen atoms existing commonly in three pyramid system. The peak at 684 cm^−1^ (B_2g_, B_3g_ symmetry) attributes to double coordinated V_2_-O stretching bond associated with the corner shared oxygen atom which exist commonly in two pyramid system. The high-frequency peak at 991 cm^−1^ is attributed to the in-phase stretching vibration of the terminal unshared oxygen V=O bond. The obtained peaks for vanadium oxide are in good agreement with other reported results, where the Raman spectra of the oxide material could be affected by deviation in oxygen stoichiometry^34^. The insert shows the Raman spectra of carbon with two broad peaks around 1584 cm^−1^ and approximately 1350 cm^−1^ which represents D and G band respectively. G band corresponds to the stretching E_2g_ vibration mode of sp^2^ hybridized carbon atoms arranged in two-dimensional hexagonal lattice, which represents the in-plane graphite Raman active modes. D band or defect band is ascribed to the sp^3^ hybridized carbon with dangling bond, which corresponds to the breathing mode of A_1g_ symmetry. The broad peak of D-band confirms the existence of amorphous carbon wrapped over the VO_2_(M) nanofiber, which supports the XRD results.

**Figure 5:**
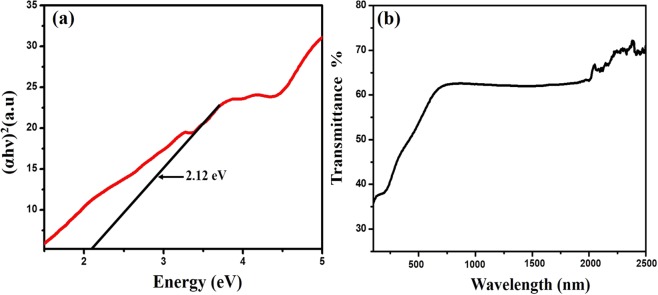
(**a**) Tauc plot for band gap measurements and (**b**) UV-Vis-NIR transmittance characterization of as-synthesized VO_2_(M)/C nanofiber.

The optical band gap of the materials can be evaluated from the absorption spectra using a most widely used Tauc plot. The relation between absorption coefficient α and photon energy hν is fitted by Equation(1):

1 $$\alpha h\vartheta =A{(h\vartheta -{E}_{g})}^{n}$$

where A is constant, E_g_ is optical bandgap to be calculated. The value n depends on the different allowed transition in the material, which equals to 2, 3, ½, 3/2 for indirect allowed, indirect forbidden, direct allowed and direct forbidden transition. The extrapolated plot in Fig. 5(a). shows that the material has a direct band gap of 2.12 eV, which is approximately close to the band gap range reported for vanadium-based oxide in the literature^35^. The optical transmittance of the as-prepared carbon coated VO_2_(M) nanofiber was studied using a UV-Vis-NIR spectrophotometer in the wavelength range from 300–2500 nm at RT. From Fig. 5(b), The result shows that the material exhibits more than 60% transmittance in the visible range at room temperature. The IR transmittance is reaching nearly 70% at 2500 nm. These results are finely matching with reported transmittance studies of VO_2_(M)^36^. The carbon present over the VO_2_(M) did not effect on the optical property of the core material.

**Figure 6:**
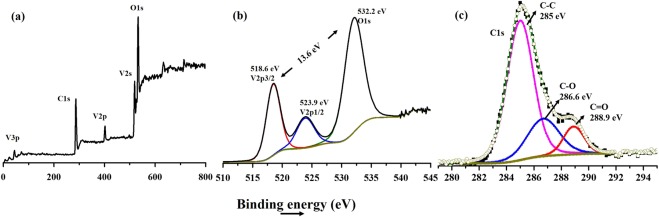
XPS spectra of VO_2_(M)/C nanofiber (**a**) overall survey scan. High resolution spectra of element (**b**) V (**c**) C.

**Figure 7:**
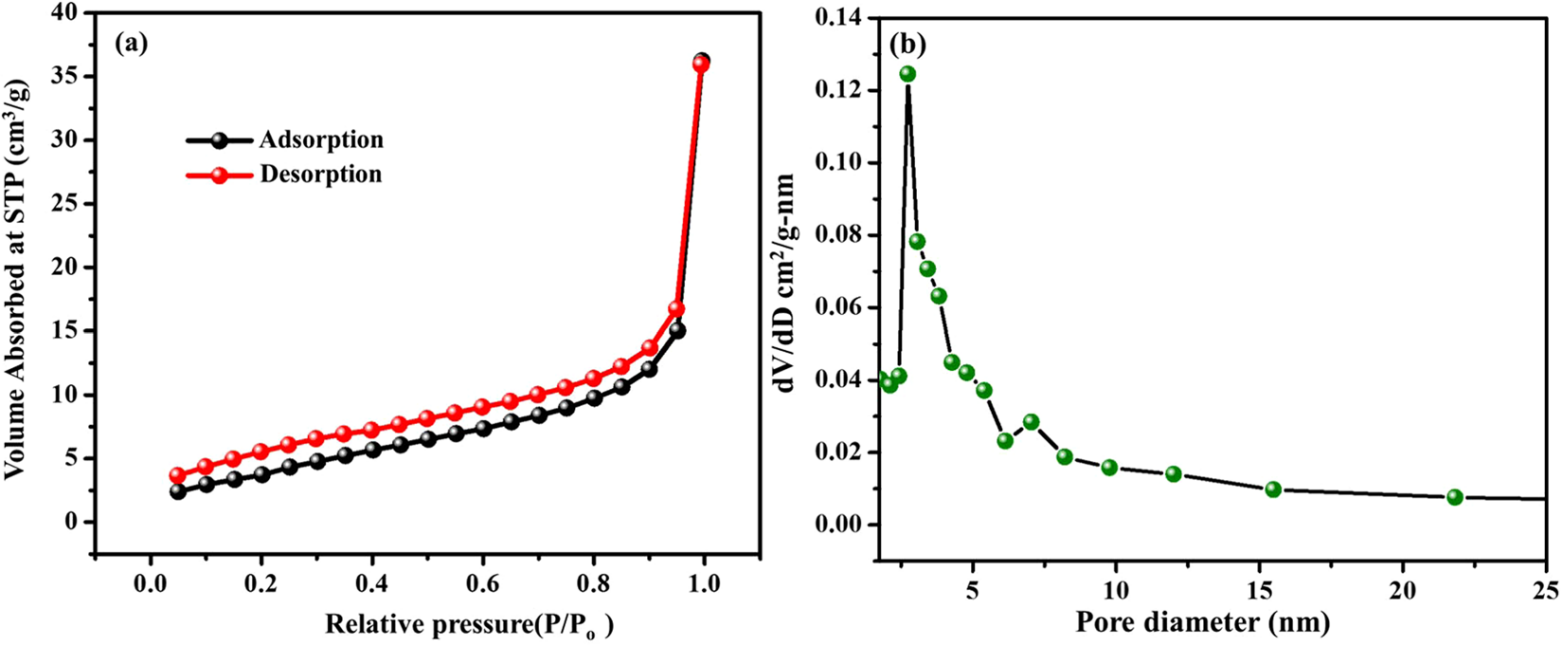
The N_2_ adsorption-desorption isotherm of VO_2_(M)/C nanofibers (**a**) BET surface area analysis (**b**) pore size distribution.

Information on elemental composition, surface electronic structure and oxidation state of the as-prepared VO_2_(M)/C nanofiber was obtained through XPS analysis. High energy spectra for C1s, V2p and O 1s were taken with the pass energy of 30 eV and step size of 0.1 eV. The XPS spectrum was calibrated by the C1s peak (284.6 eV). The element of carbon, vanadium and oxygen are detected in the survey spectra as shown in Fig. 6a. The high-resolution XPS spectra in Fig. 6b for vanadium indicates two peaks at 518.6 and 532.2 eV, which is associated with V2p3/2 and V2p1/2 spin-orbit splitting of VO_2_. A strong symmetrical peak at 532.2 eV is ascribed to O1s in VO_2_. Thus XPS result shows the formation of amorphous carbon coated VO_2_ consisting of V^4+^ and O^2−^. This evidenced the characteristic V^4+^ oxidation state of vanadium. Also from the literature, the difference in binding energy between the V2p3/2 and O1 also validates the oxidation state^33,37^. The peak at around 285 eV clearly indicates the presence of carbon, corresponding to C1s, which is attributed to the decomposition of PVP during calcination. The deconvoluted as well as their Gaussian fitting of carbon peaks are given in Fig. 6c. Brauner Emmet Teller surface area analysis of the as synthesised VO_2_(M)/C nanofiber were done to investigate the porous nature of the material using nitrogen adsorption, desorption analysis. Figure 7a, shows the isotherm curves obtained for the evaluation of surface area, which was approximately identical to Type I isotherm accompanied by type H3 hysteresis loop. The isotherm is not enclosed, which may be due to the swelling phenomenon due to the formation of very narrow slip pores or bottle shaped pores commonly present over the microporous structure. Also, there shows certain nitrogen adsorption below the relative pressure of P/P_o_ < 0.1, which evident the formation of micropores. Amorphous carbon formed over the surface may be the prompt source for the formation of the micropores. The surface area of the VO_2_(M)/C was around 15 m^2^/g. Since the material contains very narrow pores and the nitrogen molecules moves very slowly at 77 K, this limits the adsorption. The pore size distribution using the BJH method is given in Fig. 7b. Average pore radius of 1.9 nm was measured, which justifies the formation of micro pores due to the presence of carbon wrapped over the VO_2_(M) surface.

The first order phase transition properties of VO_2_(M)/C nanofiber was analysed by the change in enthalpy using DSC measurements and the curves obtained are shown in Supplementary Fig. S3. The phase transition temperature for the bulk VO_2_ is reported at around 68 °C^38^. The curve obtained for heating and cooling probes the phase transition behaviour, which is different than the normal phase transfer trend reported for VO_2_(M) pure phase. There are several possible factors like defects, doping, stress, stoichiometry and size effects, which can make an abrupt shift in this phase transition temperature^39,40^. The endothermic and exothermic transition temperature obtained for heating and cooling are 90.2 °C and 82.5 °C respectively. The phase transition temperature ${T}_{c}=({T}_{c}+{T}_{h})/2$ for the as prepared carbon coated VO_2_(M) nanofiber is calculated as 86 °C, which is much higher than the reported bulk VO_2_(M). The hysteresis width $({\rm{\Delta }}T=\,{T}_{h}-{T}_{c})$ of VO_2_(M)/C nanofiber is about 7.7 °C. Decomposition of PVP in precursor fiber added up carbon in VO_2_ nanofiber during the calcination process. As reported, the PVP used in electrospinning has the glass transition temperature around 176 °C^41^. The nanostructure size and defect due to carbon from PVP shifted the VO_2_(M) transition temperature to higher order. The amorphous nature of carbon may be the reason for the asymmetricity in endothermic and exothermic peak contribution.

**Figure 8:**
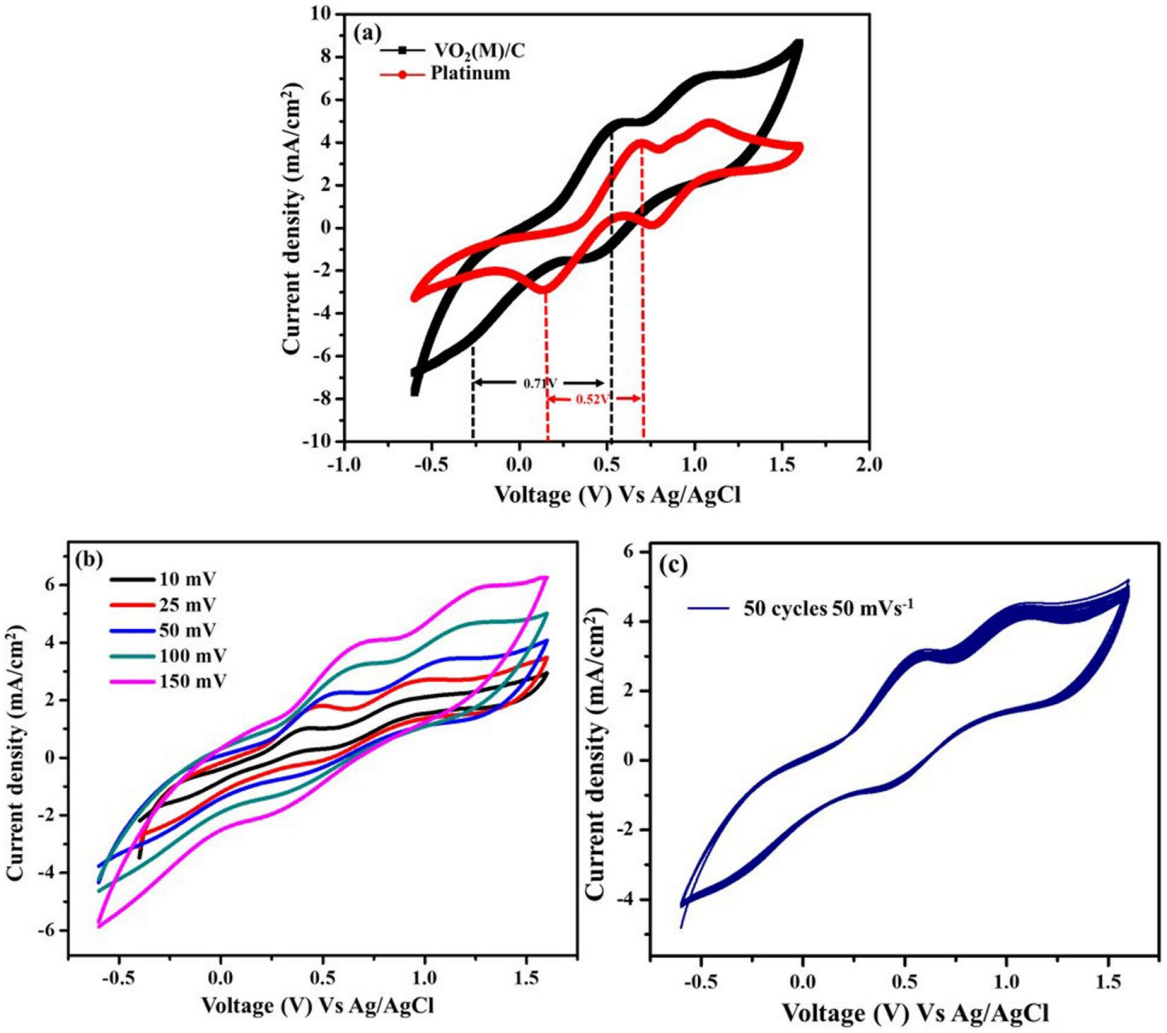
Cyclic voltammogram of (**a**) VO_2_(M)/C CE and Pt CE at a scan rate of 50 mV s^−1^ (**b**) VO_2_(M)/C at different scan rate and (**c**) VO_2_(M)/C, 50 cycle stability study at a scan rate of 50 mV s^−1^.

The electrochemical behaviour of the carbon-coated VO_2_(M) nanofiber was studied by cyclic voltammetry, electrochemical impedance and Tafel polarization. The cyclic voltammeter measurement is an effective tool to study the electrocatalytic activity towards redox process and interfacial electron transfer kinetics of the material under study. Figure 8a shows the cyclic voltammetry curve obtained for VO_2_(M)/C nanofiber in three electrode system; Ag/AgCl as reference electrode, Pt wire as counter electrode and platinum coated FTO glass or VO_2_(M)/C nanofiber coated FTO glass as a working electrode in iodide/triiodide electrolyte prepared with acetonitrile solution containing 0.1 M lithium perchlorate, 10 mM Lithium Iodide, and 1 mM Iodine and operated at a scan rate of 50 mV with the potential window from −0.6 to 1.6 V. Two typical pairs of peaks were observed from CV for both counter electrodes.

Theoretically, the peaks at negative potential correspond to the reaction

2 $${{I}_{3}}^{-}+2{e}^{-}\leftrightarrow 3{I}^{-}$$

and other peak at positive potential corresponds to the reaction

3 $$\,3{I}_{2}+2{e}^{-}\leftrightarrow 2{{I}_{3}}^{-}$$

In DSSC, the reduction reaction of I_3_^−^ will take place in the counter electrode to complete the circuit conductivity and to regenerate the dye molecules and hence it is necessary to study the oxidation and reduction of I^−^/I_3_^−^ at negative potential side. It is reported that the electrocatalytic ability of a counter electrode for an I_3_^−^ reduction in DSSC relates to the cathodic peak current at more negative potential^42^. The higher the cathodic peak current density, better the catalytic activity of the counter electrode^43,44^. The oxidation and reduction peak obtained at lower potential and higher potentials are denoted by the Equations(2,3), respectively. Counter electrode catalytic ability is estimated by two characteristic parameters such as peak current density and peak to peak separation (E_pp_). The peak to peak separation is calculated from Equation(4).

4 $${E}_{pp}=|{E}_{p}(anodic)-{E}_{p}(cathodic)|$$

From Fig. 8a, the peak to peak separation (E_pp_) for Pt CE was ~520 mV while for VO_2_(M)/C CE shows ~741 mV. Furthermore, the current density obtained for VO_2_(M)/C nanofiber electrode is higher that of Pt. The higher current density obtained for VO_2_(M)/C might result from the fibrous one-dimensional structure, porous nature, roughness over the surface and catalytic activity of the carbon, which is deposited over the VO_2_(M) fiber. Higher peak current density and E_pp_ separation indicate the good catalytic activity of the VO_2_(M)/C based counter electrodes. Figure 8b shows the CV of VO_2_(M)/C electrode at different scan rates ranging from 10 mV s^−1^ to 125 mV s^−1^ for the iodide/triiodide reduction. The shape of the CV curve, its peak position is same like Pt, which reveals that the prepared nanofibers exhibit a good electrocatalytic activity like Pt. In Fig. 8a, CV analysis shows a small peak appeared at 0.9 V. Reports explained that platinum is highly reactive towards halides and it gets absorbed on platinum both in electroactive and non-electroactive forms. This interaction results in appearance of extra peak and/or shift in the redox peak on CV analysis^45,46^. The current density increases linearly with an increasing scan rate indicating the catalytic activity of inner sites of VO_2_(M)/C nanofiber. Though VO_2_(M) is wrapped with microporous carbon, the redox activity of nanofiber is revealed from the cyclic voltammogram. Also, the electrochemical stability of VO_2_(M)/C sample was recorded for 50 consecutive cycles with the scan rate of 50 mV s^−1^ and the potential window of −0.6 to 1.6 V vs. Ag/AgCl as given in Fig. 8b. The Fig. 8c shows overlapped CV peaks and constant anodic and cathodic peak current, which reveals that the VO_2_(M)/C nanofiber possess good chemical stability, reversible redox activity and strong adhesion on the FTO substrate.

**Figure 9:**
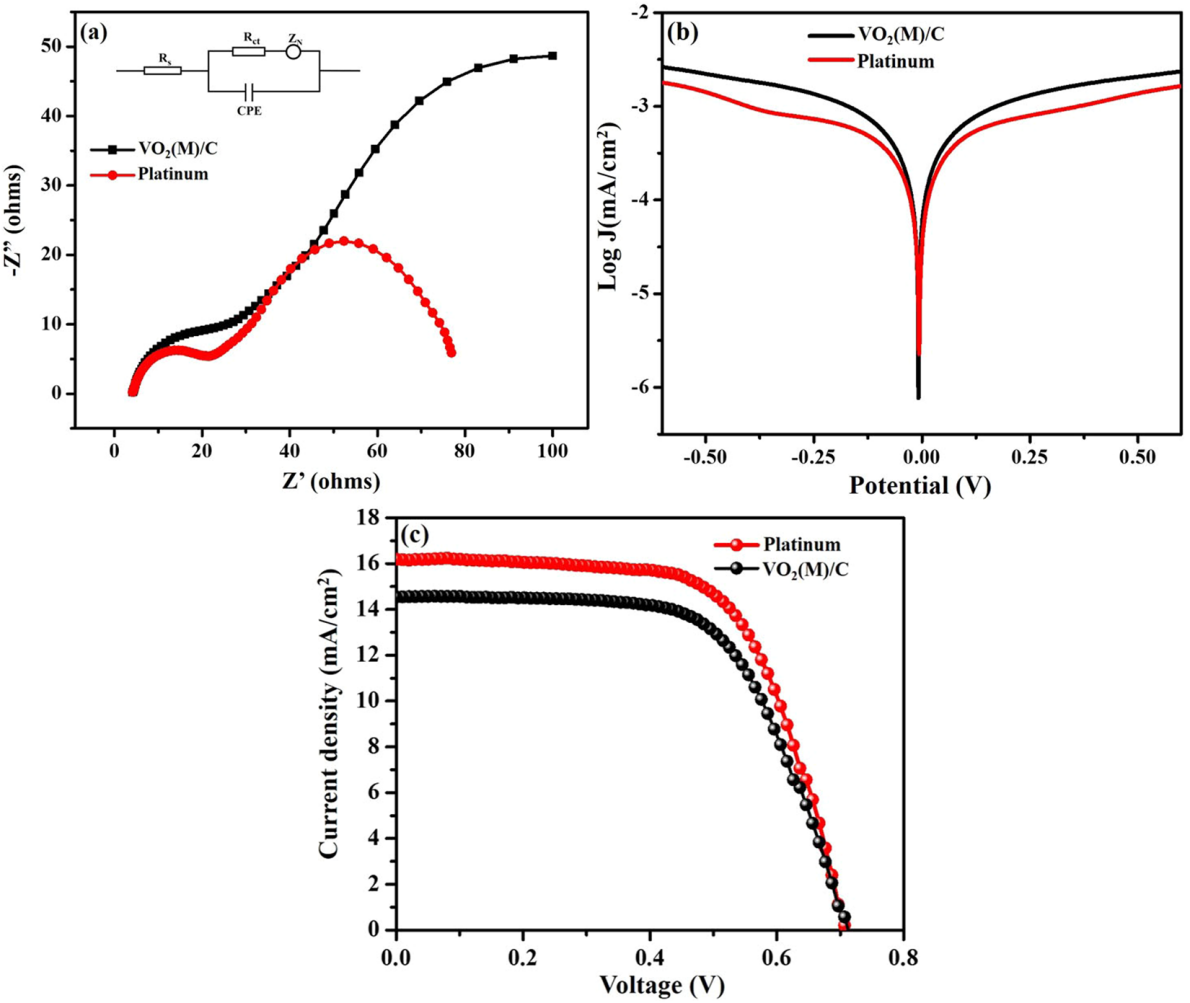
(**a**) Electrochemical Impedance spectra of VO_2_(M)/C and Pt CE in symmetric cell configuration consist of two identical CEs at 0 V under dark condition. (Inset: Equivalent circuit fitted for the data using EC-lab software). (**b**) Tafel polarization plot of symmetric cell with VO_2_(M)/C CE and Pt CE and (**c**) The characteristic DSSCs J-V photocurrent voltage graph for VO_2_(M)/C and Pt CE.

The intrinsic charge transfer and transport kinetics at the interface of electrode/electrolyte can be clearly understood by CE/electrolyte/CE symmetric cell configuration using electrochemical impedance spectroscopy technique. Figure 9a shows the Nyquist plot of Pt and VO_2_(M)/C CE. The resultant impedance data is fitted with the equivalent circuit model, which is illustrated in the insert figure. The semicircle in the high-frequency region represents the contribution of Helmholtz capacitance (CPE) and charge transfer resistance R_ct,_ which are the measures of an exchanged electron between the CE and the electrolyte interface. The diameter of the high-frequency semicircle in the Nyquist plot determines the value of R_ct_, which is the crucial parameter that can significantly influence the performance efficiency of the DSSC. The semicircle in the lower frequency region is assigned to Nernst diffusion impedance (Z_N_) of the redox couple in the electrolyte: R_s_ represents ohmic series resistance, which is the intrinsic resistance due to the combined contribution from the conductive FTO substrate resistance and lead contact resistance. The intersection of impedance response with horizontal axis stands for the value of R_s_. The values R_s_ and R_ct_ obtained from the fitted results are denoted in Table 1. Nyquist plot of VO_2_(M)/C CE depicts that the magnitude of R_ct_ is slightly higher in magnitude to that of Pt CE, which might be due to the microporous nature of the carbon wrapped nanofiber and poor interconnectivity of the short length nanofiber, which is observed from morphological analysis. Though the charge transfer resistance is slightly higher to Pt, the value of R_s_ is close to that of Pt CE. This result validates the photovoltaic performance of the device with microporous VO_2_(M)/C CE electrode comparble to Pt CE.

**Table 1:** Summary of the catalytic resistance values and their photocurrent voltage characteristic of VO_2_(M)/C and Pt.

Electrodes	*R*_*s*_(Ω/cm^2^)	*R*_*ct*_(Ω/cm^2^)	*J*_*sc*_(mA/cm^2^)	*V*_*oc*_ (V)	*FF*	*η (%)*
Platinum	4.09	17.20	16.24	0.71	64.06	7.39
VO_2_(M)/C	4.30	22.61	14.61	0.71	62.86	6.53

Tafel polarization of VO_2_(M)/C was performed to further analyse the charge transfer kinetics at the interface of CE/electrolyte using a symmetric cell configuration. The polarization curve displays the logarithmic current density (log J) as a function of voltage (V). Theoretically, the polarization curve has three distinct potential regions. The first region is a lower potential zone (|V| ≤ 120 mV), which is called as the polarization zone and second region is middle zone or tafel zone with a sharp slope, which determines the catalytic activity of the electrode. The final region is a higher potential zone (|V| > 400 mV) designated as a diffusion zone, which evaluates the electrode ion diffusion. The parameters such as exchange current density (J_0_) and limiting diffusion current density (J_lim_) can be elucidated from the polarization plot, which further validates the electrochemical performance of the electrode. In the Tafel plot, the tangent slop of cathodic or anodic branch with the equilibrium potential details the information about the exchange current density (J_0_) on the electrode. Figure 9b shows the Tafel polarization of Pt and VO_2_(M)/C CE nanofiber. The J_0_ value for Pt is higher than that of VO_2_(M)/C nanofiber, which evidence the appreciable catalytic activity of as synthesized nanofiber. Also, there was a correlation between J_0_ and R_ct_ given by Equation(5),

5 $${J}_{0}=\frac{RT}{nF{R}_{ct}}$$

where R is a gas constant, T is temperature, n is the number of electrons involved in the reaction of I_3_^−^ to I^−^ and F is the Faraday’s constant. From the above relation, the higher J_0_ value implies the lower R_ct_ contribution of the electrode. EIS measurement justifies the value of R_ct_ for Pt and VO_2_(M)/C CE. The limiting diffusion current density (J_lim_) depends on the diffusion coefficient of the redox couple in the electrolyte^47^. From Fig. 9b, the diffusion zone showed a slightly higher J_lim_ value for Pt than VO_2_(M)/C, showing its good diffusion property. These results implies that VO_2_(M)/C nanofiber catalytic performance was comparable to that of Pt.

**Figure 10:**
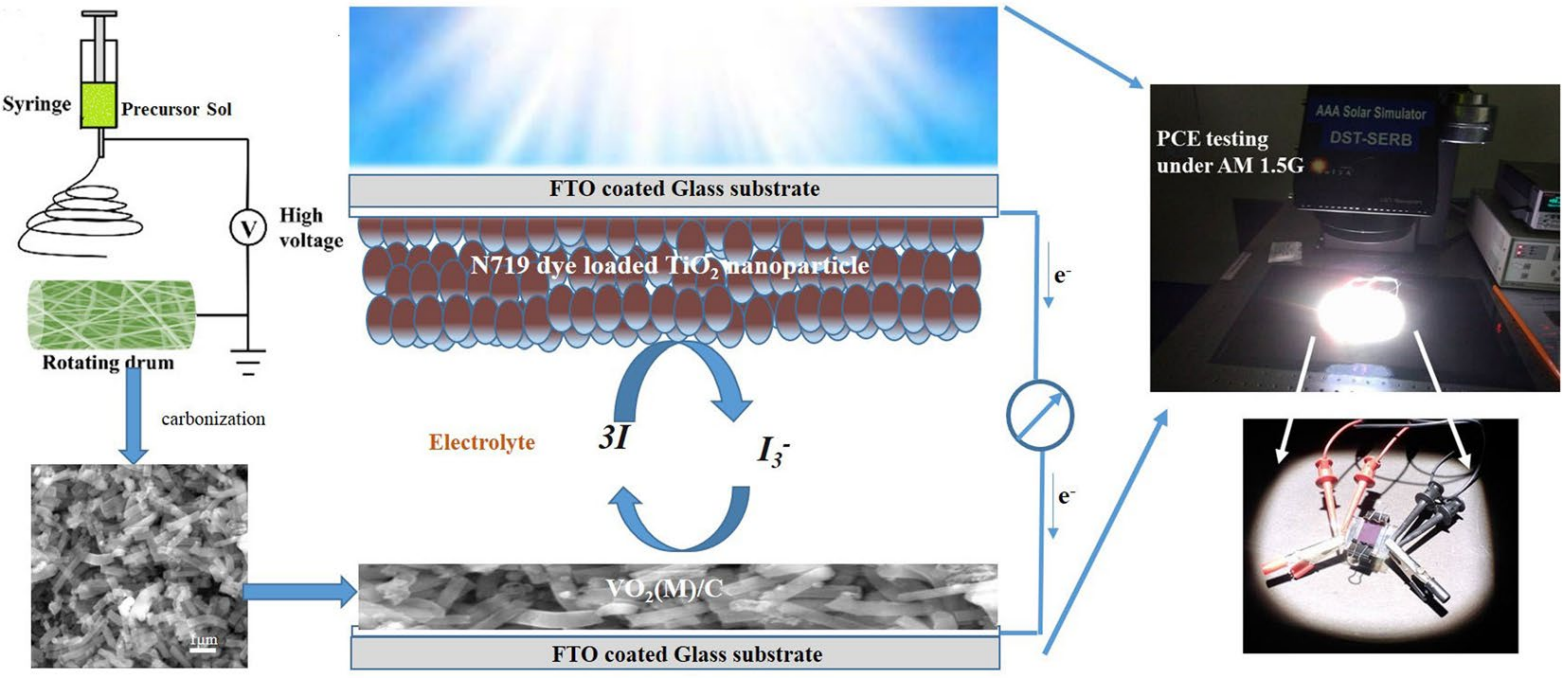
Schematic representation of as fabricated DSSC and photovoltaic testing.

The DSSCs with photoanode/electrolyte/counter electrode were fabricated and the photo current density- photovoltage (J-V) characteristics under 100 mW/cm^2^ solar irradiance with an air mass 1.5 (AM 1.5 G) global were measured. The characteristic photon generated current density-voltage (J-V) curves of the DSSC device with VO_2_(M)/C nanofiber CE, and thermally decomposed Pt CE are displayed in Fig. 9c. The derived photovoltaic parameters are tabulated in Table 1. The DSSC with VO_2_(M)/C nanofiber CE obtained efficiency η of 6.53% with 14.61 mA/cm^2^ of J_sc_, 0.71 V of V_oc_, 64.06 of FF and Pt obtained efficiency η of 7.39% with 16.24 mA/cm^2^ of J_sc_, 0.71 V of V_oc_, 62.86 of FF. Figure 10 shows the schematic representation of fabricated DSSC and photovoltaic testing. The observed power conversion efficiency value of DSSC with VO_2_(M)/C CE was much higher than that of earlier reports based on VO_2_(M) CE based DSSC which is 1.25%^21^. The improvement in V_oc_ and J_sc_ values of VO_2_(M)/C CE is due to the unique one-dimensional fibrous structure and wrapped carbon over the VO_2_(M), which improved the overall catalytic performance towards redox electrolyte.

## Conclusions

In summary, uniform VO_2_(M)/C nanofiber were successfully prepared by carbonization of the as woven electrospun nanofiber, which served as a counter electrode for dye sensitized solar cells. The structural, morphological and compositional of the as prepared material influenced the overall properties and application of the material. The electrochemical behaviour of the material were tested with cyclic voltammetry, electrochemical impedance analysis and Tafel polarization, which successfully demonstrated that the carbon-wrapped VO_2_(M) CE possess appreciable electrocatalytic activity towards iodide/triiodide redox electrolyte and low charge transfer resistance comparable to Pt CE in the cell assembly. The typical DSSC power conversion efficiency of VO_2_(M)/C nanofiber CE achieves 6.53% under 100 mV/cm^2^ at AM1.5 G where Pt CE exhibits 7.39%. Result demonstrated that VO_2_(M) nanofiber wrapped with carbon improved the electrocatalytic property VO_2_(M) and increased the photon to current conversion efficiency of the DSSC. This achievement paves the room for effective research to scale up the performance of this abundant and cost-effective material VO_2_(M) by carefully optimizing the structure and cell architecture to participate in the future energy demand.

## Supplementary information

Supplementary Information

## Data Availability

All data generated or analysed during this study are included in this published article.
