# Supplementary material for: Pt-free, low-cost and efficient counter electrode with carbon wrapped VO2(M) nanofiber for dye-sensitized solar cells
Source: Sci Rep. 2019 Mar 26;9:5177. doi: 10.1038/s41598-019-41693-1 (PMC6435790; doi:10.1038/s41598-019-41693-1)
Supplement: Supplementary file 1 — Supplementary Information [file 41598_2019_41693_MOESM1_ESM.pdf]

Supplementary Information

Pt-free, low-cost and efficient counter electrode with carbon wrapped VO_2(M) nanofiber for dye-sensitized solar cells

Subashini Gnanasekar [a], Pratap Kollu [b,c], Soon Kwan Jeong [d,*] and Andrews Nirmala Grace [a,*]

[a] Centre for Nanotechnology Research, VIT University, Vellore 632014, Tamil Nadu, India.

[b] CASEST, School of Physics, Hyderabad University, Gachibowli, Hyderabad 500046, India

[c] Newton Alumnus Researcher- the Royal Society London, Cavendish Laboratory, Department of Physics, University of Cambridge, Cambridge CB3 0HE, UK

[d] Climate Change Technology Research Division, Korea Institute of Energy Research, Yuseong-gu, Daejeon 305-343, South Korea

Corresponding author E-mail: anirmalagladys@gmail.com, anirmalagrace@vit.ac.in, jeongsk@kier.re.kr

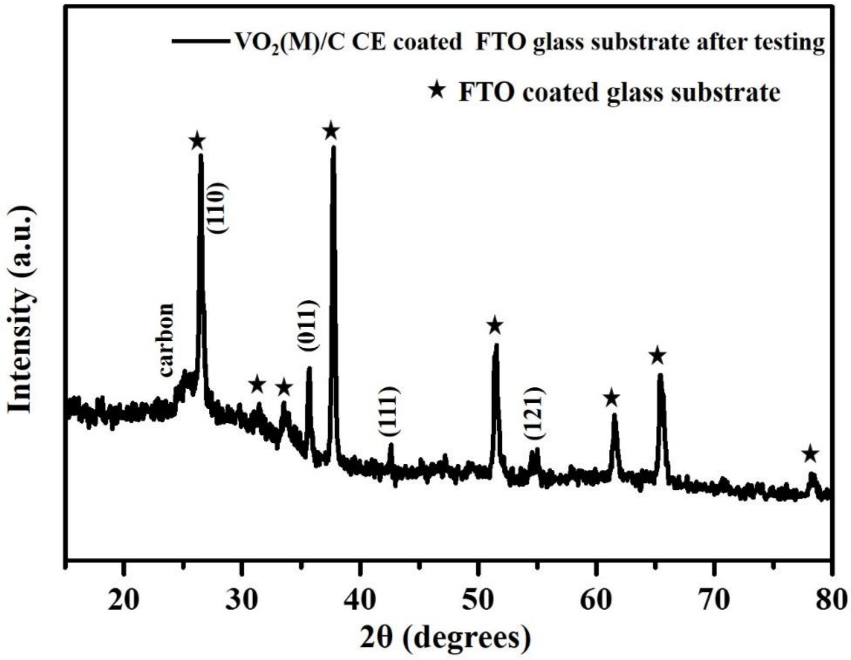

Supplementary Fig. S1. X-ray diffraction pattern of VO_2(M)/C coated FTO glass substrate after I-V testing

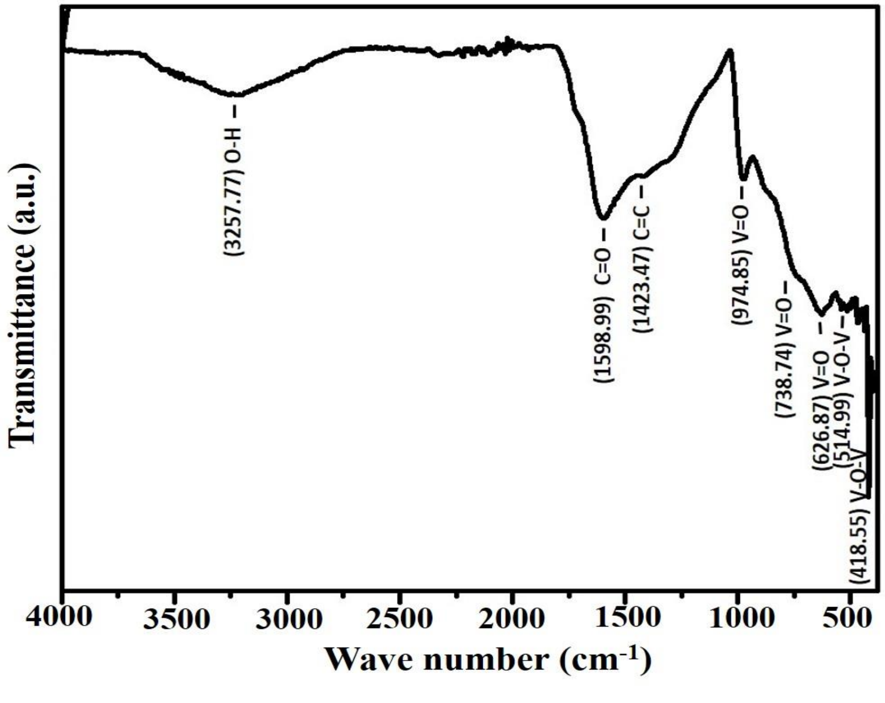

Supplementary Fig. S2. FT-IR spectra of the as-prepared VO_2(M)/C composite nanofiber

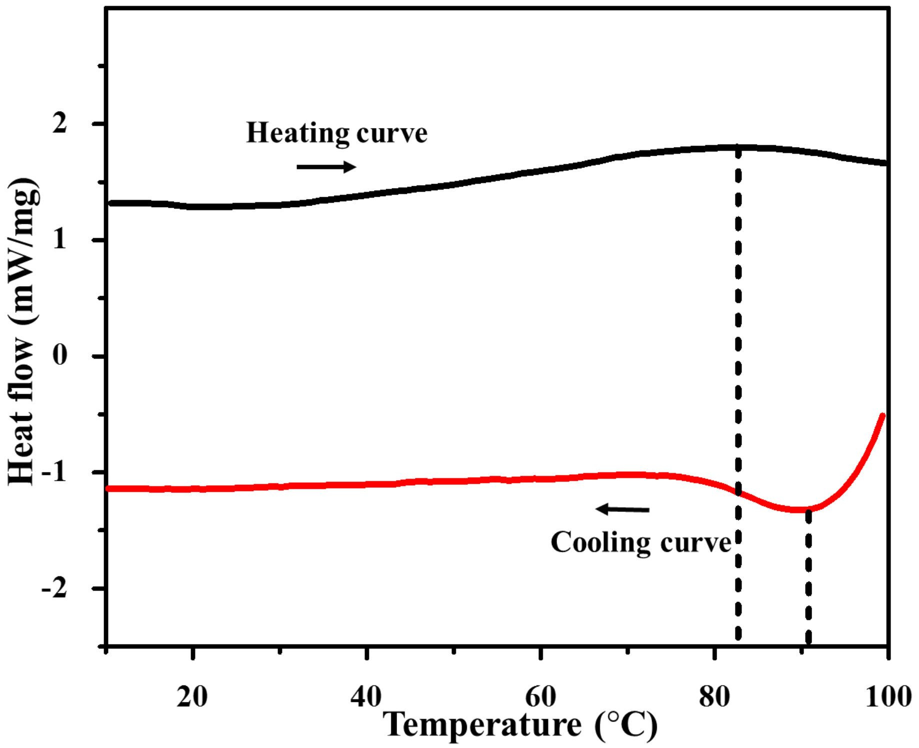

Supplementary Fig. S3. DSC of VO_2/C nanofiber with heating and cooling
